# Complete Genome Sequence of Klebsiella pneumoniae Myophage Muenster

**DOI:** 10.1128/MRA.01403-20

**Published:** 2021-01-21

**Authors:** Cody Martin, Lauren Lessor, James Clark, Tram Le, Jason J. Gill, Ryland Young, Mei Liu

**Affiliations:** a Center for Phage Technology, Texas A&M University, College Station, Texas, USA; DOE Joint Genome Institute

## Abstract

Klebsiella pneumoniae is associated with antibiotic-resistant nosocomial infections. Here, we present the annotated genome sequence of the *Klebsiella* jumbo phage Muenster. The Muenster genome sequence (346,937 bp) encodes 6 tRNAs and 561 putative protein-coding genes, including 9 tail fibers, suggesting a genetic mechanism to broaden the host range.

## ANNOUNCEMENT

Klebsiella pneumoniae is a Gram-negative opportunistic pathogen that causes severe nosocomial infections and is likely responsible for the spread of carbapenem resistance (1). Increasing drug resistance has led to the need for alternative treatment options such as phage and phage-derived treatments. We isolated and annotated the genome sequence of the *Klebsiella* phage Muenster to investigate a unique group of phages, known as jumbo phages (genomes larger than 200 kbp) (2), and assess their potential for therapy.

Muenster was isolated from wastewater collected from College Station, TX (GPS coordinates, 30.616035, −96.279732), by the soft-agar overlay method (3) and grown on carbapenem-resistant K. pneumoniae clinical isolate 44819 (GenBank accession no. NZ_NDDU00000000) aerobically at 37°C using tryptic soy medium. Transmission electron microscopy imaging performed at the Texas A&M Microscopy and Imaging Center by negative staining with 2% (wt/vol) uranyl acetate showed a myophage morphology for Muenster (4). Phage genomic DNA was prepared using a modified Promega Wizard DNA cleanup kit protocol as described previously (5). A DNA library was prepared using a TruSeq Nano kit with 300-bp inserts and then sequenced with the Illumina iSeq100 platform with paired-end 2 × 150-bp reads. The reads obtained (1,000,282 in total) were analyzed by FastQC v0.11.19 (www.bioinformatics.babraham.ac.uk/projects/fastqc) for quality control and then assembled into a single contig with 152-fold coverage using SPAdes v3.5.0 (6). The genome was closed by PCR and Sanger sequencing off the ends of the contig using a primer set (forward, 5′-GCACTATGGGTGTTGGTGGA-3′; reverse, 5′-TCAGCGTCGTCTGCATCAAA-3′). Putative protein-coding genes were annotated using GLIMMER v3 (7) and MetaGeneAnnotator v1.0 (8), and tRNAs were detected with ARAGORN v2.36 (9). Sequence homology analysis from BLASTp v2.9.0 (10) against the NCBI nonredundant (nr), Swiss-Prot, and TrEMBL databases (11), conserved folding analysis using HHpred v3.2.0 (12), and conserved domain analyses using InterProScan v5.33 (13) and TMHMM v2.0 (14) were used to assign the putative gene functions. TransTermHP (15) was used to predict the rho-independent terminators. Minus HHpred, all tools were accessed in the CPT Galaxy-Apollo interface (https://cpt.tamu.edu/galaxy-pub) (16–18). Default settings were used for all analyses.

Muenster has a 346,937-bp genome sequence with 561 putative protein-coding genes and 6 tRNAs for a 91% coding density. The genome has a 31.9% GC content, which is significantly lower than that of its host at ∼57.7% (19). The genome sequence was reopened to reflect a 20,030-bp direct terminal repeat predicted by PhageTerm (20). Genome-wide sequence similarity analysis using progressiveMauve v2.4 (21) showed that Muenster is most closely related to other *Klebsiella* jumbo phages, K64-1 (GenBank accession no. AB897757) and vB_KleM-RaK2 (JQ513383), with ∼90% nucleotide sequence similarity. This suggests that Muenster belongs to the genus *Alcyoneusvirus*. Four hundred forty-six genes encoded hypothetical proteins. Thirty-six coding DNA sequences (CDSs) had significant similarity to T4 proteins with annotated functions, and five more had high-probability HHpred scores for T4 proteins. The Muenster genome sequence has a 20,030-bp direct terminal repeat, but its large terminase is closely related to T4 gp*17*, despite the T4 packaging having a headful-packaging mechanism. Muenster encodes nine putative tail fibers, most of which are followed by transcriptional terminators. This suggests that the phage uses a controlled genetic mechanism to broaden the host range by modifying the tail fibers (22).

### Data availability.

The genome sequence of phage Muenster was deposited under GenBank accession no. MT708547 and BioSample accession no. SAMN14646297. The BioProject accession no. is PRJNA222858, and the SRA accession no. is SRR11575707.
